# Enhanced electron transfer mediated detection of hydrogen peroxide using a silver nanoparticle–reduced graphene oxide–polyaniline fabricated electrochemical sensor†

**DOI:** 10.1039/c7ra11466d

**Published:** 2018-01-04

**Authors:** Vijay Kumar, Rajeev Kumar Gupta, Ravi Kumar Gundampati, Devendra Kumar Singh, Sweta Mohan, Syed Hadi Hasan, Manisha Malviya

**Affiliations:** Nanomaterial Research Laboratory, Department of Chemistry, Indian Institute of Technology (BHU) Varanasi-221005 U.P. India vijuevs@gmail.com +91-542-6702861 +91 9839089919; Fuel Cell Technology, Department of Chemistry, Indian Institute of Technology (BHU) Varanasi-221005 U.P. India shhasan.apc@itbhu.ac.in; Department of Chemistry and Biochemistry, University of Arkansas Fayetteville Arkansas-72701 USA

## Abstract

The current study aims at the development of an electrochemical sensor based on a silver nanoparticle–reduced graphene oxide–polyaniline (AgNPs–rGO–PANI) nanocomposite for the sensitive and selective detection of hydrogen peroxide (H_2_O_2_). The nanocomposite was fabricated by simple *in situ* synthesis of PANI at the surface of rGO sheet which was followed by stirring with AEC biosynthesized AgNPs to form a nanocomposite. The AgNPs, GO, rGO, PANI, rGO–PANI, and AgNPs–rGO–PANI nanocomposite and their interaction were studied by UV-vis, FTIR, XRD, SEM, EDX and XPS analysis. AgNPs–rGO–PANI nanocomposite was loaded (0.5 mg cm^−2^) on a glassy carbon electrode (GCE) where the active surface area was maintained at 0.2 cm^2^ for investigation of the electrochemical properties. It was found that AgNPs–rGO–PANI–GCE had high sensitivity towards the reduction of H_2_O_2_ than AgNPs–rGO which occurred at −0.4 V *vs.* SCE due to the presence of PANI (AgNPs have direct electronic interaction with N atom of the PANI backbone) which enhanced the rate of transfer of electron during the electrochemical reduction of H_2_O_2_. The calibration plots of H_2_O_2_ electrochemical detection was established in the range of 0.01 μM to 1000 μM (*R*^2^ = 0.99) with a detection limit of 50 nM, the response time of about 5 s at a signal-to-noise ratio (S/N = 3). The sensitivity was calculated as 14.7 μA mM^−1^ cm^−2^ which indicated a significant potential as a non-enzymatic H_2_O_2_ sensor.

## Abstract 2

The current study aims at the development of an electrochemical sensor based on a silver nanoparticle–reduced graphene oxide–polyaniline (AgNPs–rGO–PANI) nanocomposite for the sensitive and selective detection of hydrogen peroxide (H_2_O_2_).

## Graphical abstract

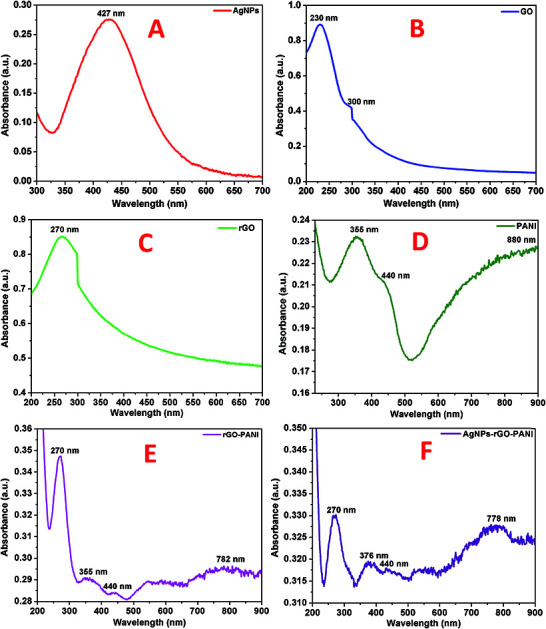

## 1. Introduction

Hydrogen peroxide (H_2_O_2_) has broad industrial applications and vital significance in various environmental, biological, chemical, pharmaceutical and clinical processes.^1,2^ It is created during oxidative stress in cells, the concentration of which may be used as a primary indicator of reaction. In addition to this, H_2_O_2_ is also produced as a by-product of several enzyme-catalysed reactions such as cholesterol oxidase, glucose oxidase, glutamate oxidase, lactate oxidase *etc.*^3^ Therefore, the development of simple and cost effective analytical techniques for the selective and quantitative detection of H_2_O_2_ has become an immense challenge after the researcher engaged in the detection of H_2_O_2_.^4^ So, far several analytical techniques such as such as spectrophotometry.^5^ Fluorimetry,^6^ electrochemical^7^ and chemiluminescence^8^ have been employed for the determination of H_2_O_2_. Among these, the electrochemical method offers a rapid and simple approach for the sensitive detection of H_2_O_2_. The electrochemical method is performed through enzyme and non-enzyme based biosensor. Although, the enzyme-based sensors so far developed, are simple with high sensitivity and selectivity but the instability, high initial cost, complex immobilisation procedure and shorter lifetime has limited the practical applicability in biosensing applications.^9^ For avoiding these problems, several studies have been made out to develop non-enzyme based biosensors having a high and broad range of response.

The development of nanoscience and nanotechnology has offered new avenues for the application of carbon-based nonmaterial such as graphene,^10^ carbon nanotube,^11^ porous carbon^12^*etc.* in electrochemical analysis. Graphene is a sp^2^ hybridized two-dimensional sheet of single-atom thick carbon which possesses extraordinary chemical, electrical, mechanical properties, rapid electron transfer kinetics and great electrocatalytic characteristics.^13^ Due to these characteristics graphene is being widely used in the synthesis of nanocomposite for sensing applications. The graphene could be prepared by several procedures among which the reduction of GO may be a very promising approach. The reduction of GO could be performed by several ways such as thermal reduction,^14^ electrochemical reduction^15^ and chemical reduction.^16^ Among these, the reduction of GO by chemical route is the most widely used route for obtaining reduced graphene oxide (rGO). But the major drawback of the rGO is agglomeration and reverting to graphite. To overcome these problems, at present the researchers are focused on the preparation of the nanocomposite of rGO with conducting polymers which provide stability to it by combining with the oxygenated functional groups. Therefore, new polymer nanocomposite with superior conducting properties can be obtained by surface coating of the rGO with conducting polymer due to a synergistic effect. This effect facilitates the shorter ion diffusion path with π–π interaction which enhances the transport of electron. Among several conducting polymers, polyaniline (PANI) is considered as a perfect conducting polymer which has attracted the interest of the researchers because of its low cost, light weight, environmentally good, high energy density, controllable electrical conductivity, and faster loading/deloading rate during charge/discharge process.^17,18^ But, the lower processing ability and weaker mechanical strength of PANI and its sensor could be enhanced by fabricating with metal nanoparticles (MNPs). The MNPs–rGO–PANI can modify the transfer of an electron to a greater extent which owed to the strong electronic interaction among MNPs, rGO, and PANI. Several nanocomposites containing MNPs, rGO, and PANI have been prepared which corroborated the enhanced electrocatalytic activity as compared to pure graphene, PANI, and MNPs. For example, Zheng *et al.*, 2016 has prepared the CuNPs/graphene/PANI nanocomposite for highly sensitive electrochemical sensing of glucose.^19^ Ghanbari & Moloudi, have detected uric acid and dopamine using ZnO/rGO/PANI nanocomposite.^20^

In the current work, AgNPs–rGO–PANI nanocomposite was prepared to detect H_2_O_2_ by an electrochemical technique where the rGO was first modified with PANI to avail the hydrophilic functional –NH_2_ groups which facilitated the decoration of AgNPs on rGO–PANI and formed AgNPs–rGO–PANI nanocomposite.

## 2. Experimental

### 2.1 Chemicals

The graphite powder (mesh size 150 mm), phosphoric acid (H_3_PO_4_), sulfuric acid (H_2_SO_4_) and potassium permanganate (KMnO_4_) were procured from Sigma-Aldrich. Hydrazine (NH_2_NH_2_) and ammonium hydroxide solution (28–30% NH_3_ basis) were purchased from SD Fine Chemicals. Aniline, AgNO_3_, ammonium persulphate (APS), hydrogen peroxide (H_2_O_2_, 30%) were procured from Sigma-Aldrich. Sodium phosphate (NaH_2_PO_4_ and Na_2_HPO_4_), potassium chloride (KCl) and acetone were procured from Merck, India whereas Nafion solution was procured from Alfa Aesar. The deionized double distilled water (DW) was used for making solutions in all the experiments.

### 2.2 Synthesis of graphene oxide (GO)

**Scheme 1:**
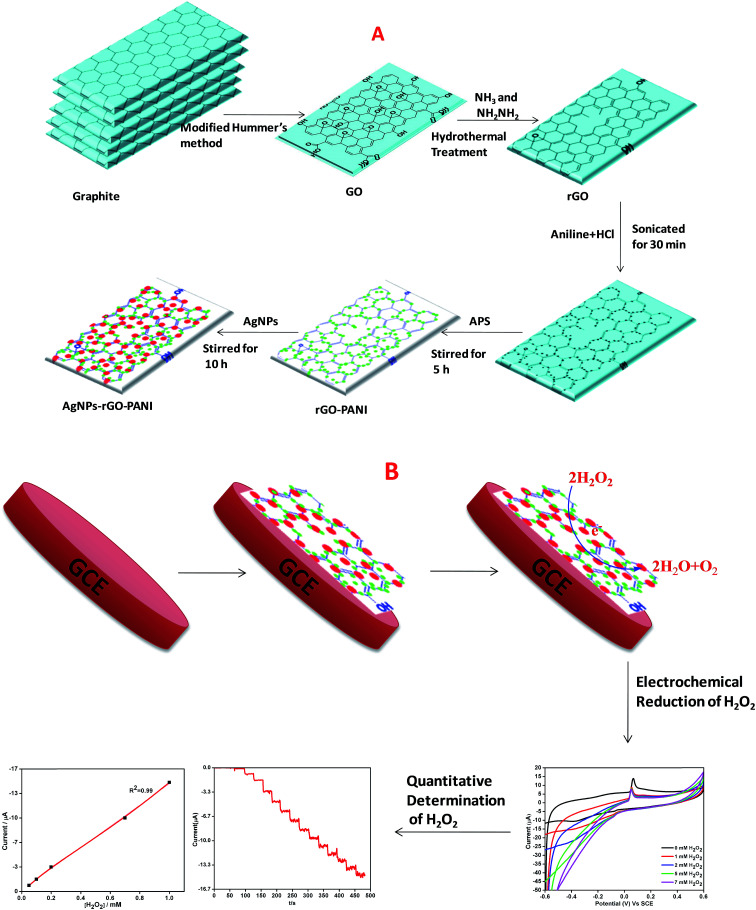
Schematic representation of preparation of (A) AgNPs–rGO–PANI nanocomposite, (B) AgNPs–rGO–PANI modified glassy carbon electrode.

The GO was prepared according to our previously reported works.^21,22^ Briefly, 3 g graphite powder was mixed into the mixture of concentrated H_2_SO_4_/H_3_PO_4_ (360 : 40 mL) then, 15 g KMnO_4_ was poured slowly into the reaction mixture. Thus, obtained final reaction mixture was continuously stirred at 50 °C for 12 h which was followed by the cooling at room temperature and poured into 1 L of DW by keeping it on the ice bath. Then 3 mL of H_2_O_2_ (30%) was added with continuously stirring for additional 2 h. After that, the reaction product was collected by centrifugation at 10 000 rpm for 15 min. Further, the obtained material was collected and followed by several times washing with 5% HCl and then many times with DW. Then final washed solid material *i.e.* GO was placed in the vacuum oven at 70 °C for 24 h. Thus obtained powdered GO was suspended in DW (1 mg mL^−1^) and well dispersed using ultrasonication for 1 h and then centrifuged (Remi, R 24) at 10 000 rpm for 10 min to avoid the unexfoliated GO. The brown colored GO suspension was reduced hydrothermally using ammonia solution (NH_3_) and hydrazine (NH_2_NH_2_) as a reducing agent. Briefly, the pH of the suspension was maintained to 10 using ammonia solution which was followed by the addition of N_2_H_4_ and stirred for 10 min. Further, the suspension was transferred to an autoclave (Teflon-lined) and kept at 200 °C for 5 h. The final black colored suspension thus obtained was collected by centrifuging several times with DW (Scheme 1A).

### 2.3 Synthesis of AgNPs–rGO–PANI nanocomposite

The schematic diagram of AgNPs–rGO–PANI nanocomposite preparation is shown in Scheme 1A. Firstly, the rGO–PANI nanocomposite was synthesized by using 1 : 100 mass ratio of graphene to aniline. For this 1.0 mg rGO and 100.0 mg aniline were taken and added to 20 mL of 2.0 mol L^−1^ HCl solution and sonicated for 30 min. After that, 10 mL aqueous APS solution (0.04 g mL^−1^) was added slowly into the above mentioned solution. After this, the solution was followed by continued stirring for 5 h wherein it turned to dark green which indicated the synthesis of the rGO–PANI nanocomposite. After stirring, the rGO/PANI nanocomposite thus obtained was centrifuged at 10 000 rpm for 15 min and washed with distilled water. The above process was repeated four times for the removal of uncoordinated impurities and thereafter dried at 60 °C.

The nanocomposite of three component system (AgNPs–rGO–PANI) was prepared by a self-assembly method. For this, 0.01 g rGO–PANI nanocomposite was added into the 25 mL colloidal AgNPs obtained through our previously reported work.^23^ Further, the reaction was stirred for next 10 h. After this, the final AgNPs–rGO–PANI nanocomposite was centrifuged at 10 000 rpm for 15 min and washed several times with distilled water and dried at 60 °C.

### 2.4 Fabrication of AgNPs–rGO–PANI on GCE

The electrochemical detection of H_2_O_2_ was performed by AgNPs–rGO–PANI modified glassy carbon electrode (AgNPs–rGO–PANI–GCE) which was prepared by deposition of ethanol–water (2 : 1) suspension of AgNPs–rGO–PANI on GCE. After drying at room temperature, the AgNPs–rGO–PANI on GCE, 15 μL of 1% Nafion solution was drop cast over it to enhance its adherence. The loading of electrochemical sensor mass was maintained 0.5 mg cm^−2^ on GCE. The GCE was found to be possessed 0.2 cm^2^ active surface.^24^ The effect of PANI on the enhanced electrocatalytic performance on H_2_O_2_ reduction was compared with an electrode without PANI (AgNPs–rGO–GCE) prepared by the same procedure. All the experiments and measurements were conducted at room temperature (Scheme 1B).

### 2.5 Electrochemical detection of hydrogen peroxide (H_2_O_2_)

CV and amperometric response of AgNPs–rGO, AgNPs–rGO–PANI–GCE was performed in 0.1 M PBS solution containing 0.5 M KCl at pH 7. The amperometric response of AgNPs–rGO–PANI–GCE in PBS in the presence of H_2_O_2_ at pH 7 led to the appearance of the response current at −0.4 V. All the CV and amperometric response experiments related to the H_2_O_2_ detection were carried out in N_2_-medium. The electrochemical impedance spectroscopy (EIS) was performed by applying the frequency in the range of 0.01–10^5^ Hz at open cell potential.

The electrocatalytic reduction of H_2_O_2_ with an AgNPs–rGO system has been studied extensively due to its similar behavior with artificial peroxidase.^25,26^ However, the detection of a precise cathodic current by examining the contribution of the PANI in the AgNPs–rGO nanocomposite plays an essential role that allows the selective detection of preferred analytes in several practical applications. In the current work, the AgNPs–rGO–PANI composite modified GCE was employed as a simple electrochemical sensor for the rapid determination of H_2_O_2_ cathodically, through the involvement of PANI in AgNPs–rGO–PANI nanocomposite.

### 2.6 Characterization

The UV-visible spectrophotometer (Evolution 201, Thermo Scientific) was used for preliminary study of AgNPs in the range of 300 to 800 nm. The involvement of various functional groups was investigated using Fourier transform infrared spectrophotometer (FTIR, Perkin Elmer Spectrum 100) in the range of 4000–400 cm^−1^. The X-ray Diffractometer (Rigaku Miniflex II) having Cu Kα radiation source and Ni filter was applied to investigate the crystallinity of the synthesized AgNPs in the range of 20° to 80° and at a scanning rate of 6° min^−1^ with 0.02° of step size. Primarily, the size and shape of the AgNPs was investigated using Field Emission Scanning Electron Microscopy with Energy-Dispersive X-ray (FE-SEM-EDX, Hitachi H-7100). The elemental speciation and the purity were confirmed by EDX analysis. The Transmission Electron Microscopy (TEM, TECNAI 20 G2) at accelerating voltage 200 kV was used to confirm the shape and size of the biosynthesized AgNPs. The Selected Area Electron Diffraction (SAED) demonstrated the concentric diffraction rings which also confirmed the crystallinity of the synthesized AgNPs. The X-ray Photoelectron Spectroscopy (XPS, AMICUS, Kratos Analytical, A Shimadzu) with Mg Ka (1253.6 eV) radiation as an X-ray source was conducted for the speciation of the nanocomposite.

The electrocatalytic activity of AgNPs–rGO–GCE and AgNPs–rGO–PANI–GCE was investigated by an electrochemical technique. Cyclic voltammetry (CV), amperometric determination and electrochemical impedance spectroscopy (EIS) were performed on a three electrode system assembly in the single chamber Pyrex glass cell. Potentiostat/galvanostat (Biologic M150) was used to take electrochemical measurements. A platinum foil (8 cm^2^) was used as a counter electrode, saturated calomel electrode (SCE) as a reference electrode and GCE as a working electrode.

## 3. Results and discussion

### 3.1 Characterization

**Fig. 1:**
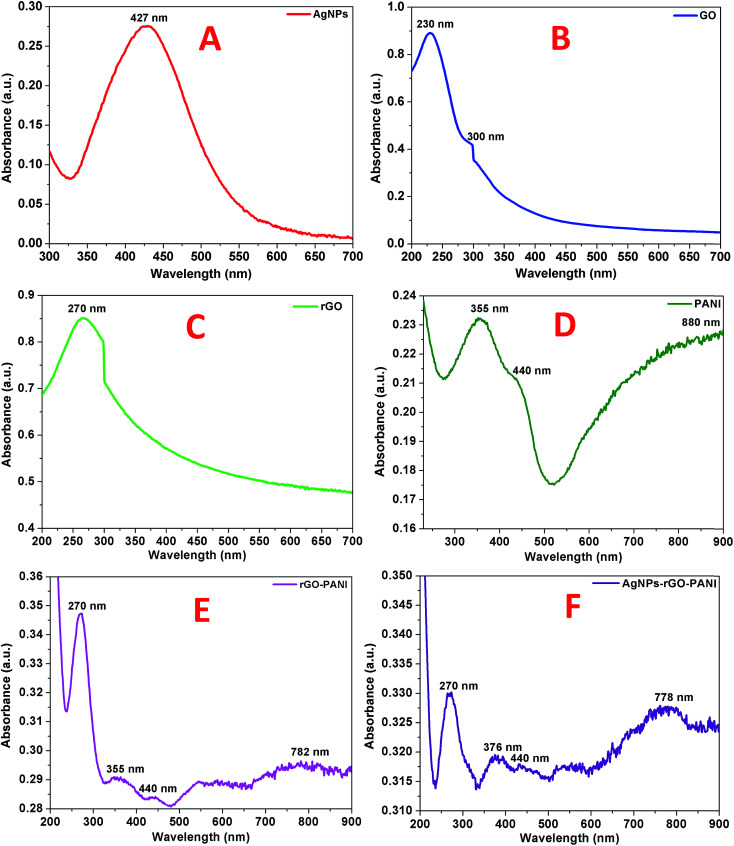
UV-visible spectra of (A) AgNPs, (B) GO, (C) rGO, (D) PANI, (E) rGO–PANI, and (F) AgNPs–rGO–PANI.

The primary confirmation of the formation of AgNPs, GO, rGO, PANI, rGO–PANI and AgNPs–rGO–PANI nanocomposite was performed by UV-visible spectroscopy which are shown in Fig. 1A–F. The spectra of AgNPs revealed a sharp peak at 427 nm which was due to its characteristic surface plasmon resonance (SPR) with *λ*_max_ in the range of 400–500 nm (ref. 27) (Fig. 1A). The UV-visible spectra shown in Fig. 1B represented the characteristic peak of GO at 230 nm and 300 nm which corresponded to the π–π* transition of C=C and n–π* transition of C=O respectively.^28^ The absorption spectra of rGO shown in Fig. 1C corresponded to the π–π* transition of C=C bond with a red shift to 270 nm which clearly indicated the reduction of GO into rGO as well as restoration of C=C bond in the rGO sheet.^29^ The UV-visible spectra of PANI shown in Fig. 1D revealed the presence of three peaks at 355 nm, 440 nm, and 890 nm which corresponded to π–π* transition, polaron, and bipolaron band transition of PANI. These three peaks confirmed the presence of a protonated emeraldine salt form of PANI.^30,31^ The peak at 270 nm, 355 nm, 440 nm and 782 nm confirmed the grafting of PANI at the sheets of rGO (Fig. 1E). Fig. 1F corroborated the UV-visible spectra of AgNPs–rGO–PANI where the peak of PANI present at 355 nm and 782 nm shifted to 376 nm and 778 nm respectively. The shifting in these peaks showed the interaction of AgNPs with the PANI grafted at the surface of rGO to form AgNPs–rGO–PANI nanocomposite.

**Fig. 2:**
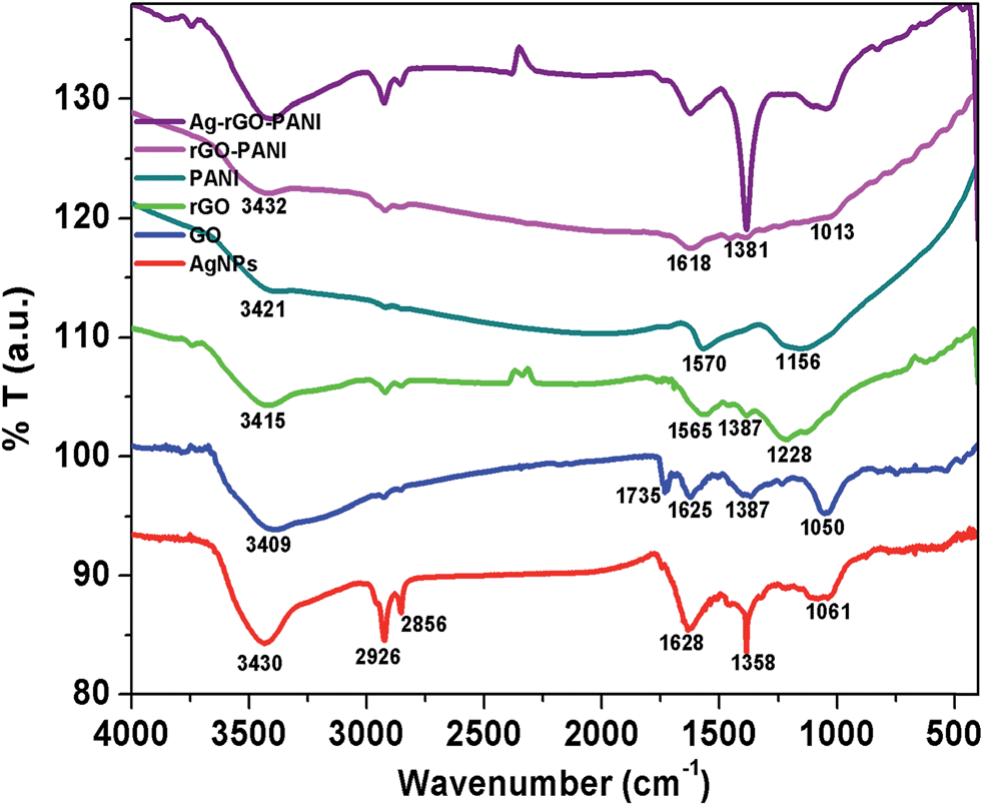
FTIR spectra showing the various functional groups present in AgNPs, GO, rGO, PANI and their involvement in the preparation of rGO–PANI, and AgNPs–rGO–PANI nanocomposite.

The FTIR spectra were recorded to investigate the presence of different functional groups in AgNPs, rGO and PANI as well as their interaction in rGO–PANI, and AgNPs–rGO–PANI nanocomposite which is shown in Fig. 2. The FTIR spectrum of green synthesized AgNPs showed the absorption band at 3430 cm^−1^, 2926 cm^−1^, 2858 cm^−1^, 1628 cm^−1^, 1385 cm^−1^, and 1061 cm^−1^. The band at 3430 cm^−1^, 2926 cm^−1^ and 2858 cm^−1^ were due to the presence of stretching vibration (*ν*_s_) of OH, C=C–H, and C=C respectively. Whereas the band at 1628 cm^−1^, 1385 cm^−1^, and 1061 cm^−1^ were due to the presence of *ν*_s_ of C=C, C=C and O–C respectively which were same as that of peaks present in tannic acid and aqueous extract of *Croton bonplandianum* (AEC) as shown in Fig. S1.† This corroborated the involvement of tannin in the AgNPs biosynthesis. The FTIR spectrum of GO revealed the presence of characteristic bands at 3409 cm^−1^ (*ν*_s_ of OH), 2927 cm^−1^ (*ν*_s_ of sp^2^ hybridized C–H), 2852 cm^−1^ (*ν*_s_ of sp^3^ hybridized C–H), 1735 cm^−1^ (*ν*_s_ of C=O carbonyl), 1665 cm^−1^ (*ν*_s_ of C=C), 1374 cm^−1^ (*ν*_d_ of C–OH), and 1050 cm^−1^ (*ν*_s_ of epoxide). Whereas the FTIR spectrum of rGO revealed the development of a new peak at 1565 cm^−1^ (sp^2^ hybridized C=C) and disappearance of peak at 1050 cm^−1^. The disappearance of 1050 cm^−1^ (*ν*_s_ of epoxide), as well as appearance of a peak at 1565 cm^−1^ (sp^2^ hybridized C=C), confirmed the successful reduction of GO to rGO. The FTIR spectrum of PANI represented the absorption bands at 3421 cm^−1^, 2923 cm^−1^, 2853 cm^−1^, 1570 cm^−1^, and 1156 cm^−1^. The band present at 3421 cm^−1^ and 2923 cm^−1^ and 2853 cm^−1^ were due to N–H *ν*_s_ of PANI and aromatic sp^2^*ν*_s_ of C–H. The band present at 1570 cm^−1^ and 1156 cm^−1^ attributed to C=C stretching deformation of the quinoid ring and aromatic C–H inplane bending.^32^ These characteristics bands confirmed the existence of emeraldine salt phase of PANI.^33^ The FTIR spectrum of rGO–PANI showed the characteristics bands of rGO and PANI at 3432 cm^−1^ (*ν*_s_ of NH), 2923 cm^−1^ (*ν*_s_ of C–H) 1618 cm^−1^ (*ν*_s_ of C=C), 1381 cm^−1^ (*ν*_s_ of C–N), and 1013 cm^−1^ (*ν*_s_ of C=C). The presence of these bands revealed the successful coating of PANI at the sheets of rGO. The observed bands of AgNPs at 862 cm^−1^ attributed N=C stretching of an aromatic amine and metal interaction which advocated the adherence of AgNPs with PANI deposited at the sheets of rGO. Such an interaction of PANI, a sheet of rGO and AgNPs confirmed the formation of AgNPs–rGO–PANI nanocomposite.

**Fig. 3:**
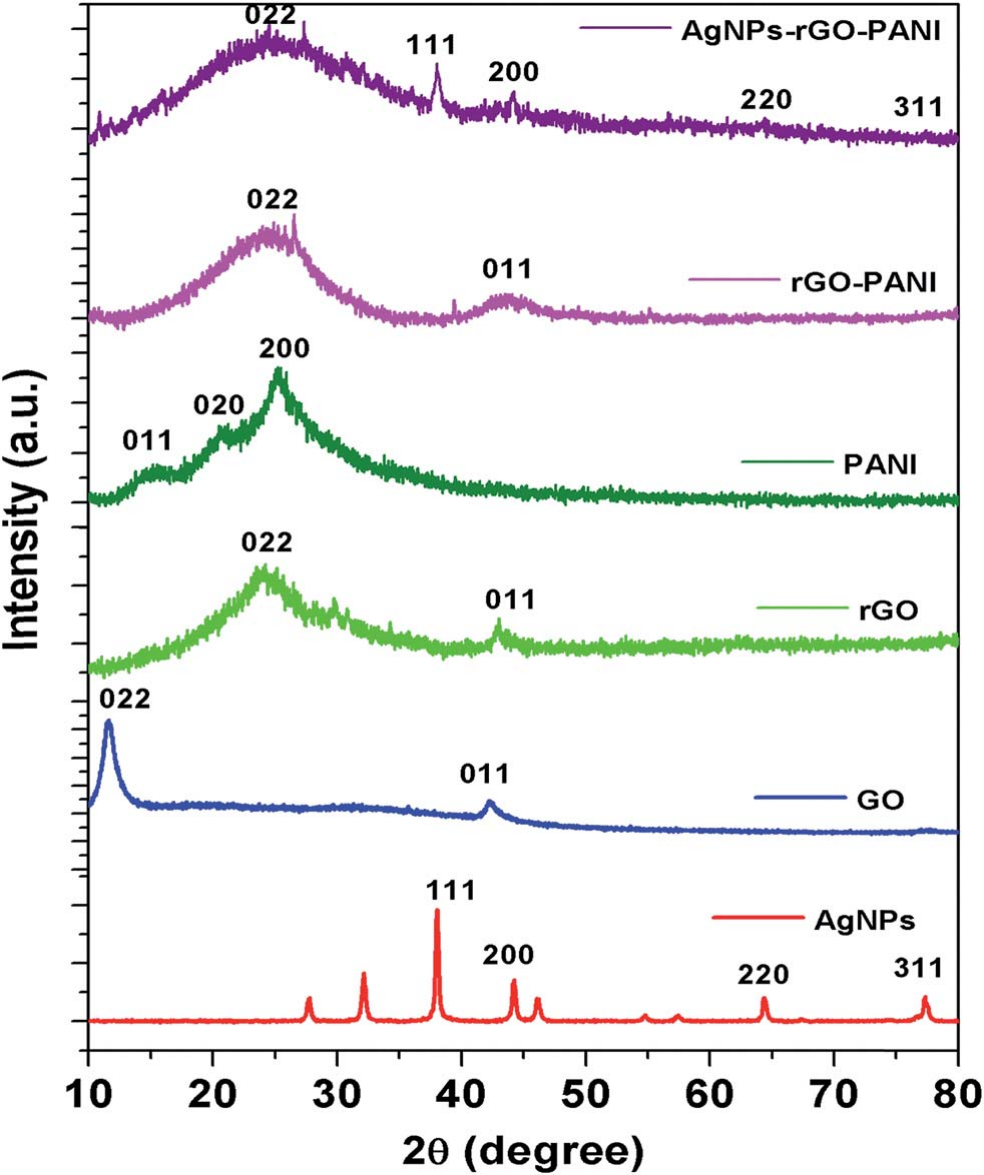
XRD spectra are showing the characteristics peak of AgNPs, GO, rGO, PANI, rGO–PANI, and AgNPs–rGO–PANI nanocomposite.

To further investigate the crystalline nature of the synthesized AgNPs, rGO, PANI and formation of rGO–PANI and AgNPs–rGO–PANI nanocomposite, XRD analysis was carried out which is shown in Fig. 3. The XRD data were obtained in the angular range 20° ≤ 2*θ* ≤ 80° at 6° min^−1^ of scanning rate and 0.02° of step size. The XRD spectrum of AgNPs depicted the diffraction peaks at 2*θ* = 38.09°, 44.04°, 64.26°, and 77.26°. These peaks were matched with the JCPDS file no. 040783 for the standard diffraction data of AgNPs which attributed to the (111), (200), (220) and (311) Bragg's reflections. These Bragg's reflections advocated the crystalline planes of metallic silver with face-centered cubic (fcc).^34^ The XRD spectrum of GO revealed the most apparent diffraction peak centred at 2*θ* = 10.9° which corresponded to the 002 Bragg's reflections with significantly expanded *d*-spacing of 0.83 nm comparatively 0.34 nm for pristine graphite.^21^ The comparatively larger *d*-spacing of rGO than pristine graphite advocated the intercalation of different oxygen bearing functional groups and water molecules between the graphitic layers. After reduction, the diffraction peak observed at 2*θ* = 10.9° disappeared and a broad peak centered at 2*θ* = 23.9°, and 43.0° was observed which attributed to (002) and (011) Bragg's reflections respectively with *d*-spacing of 0.418 nm. The decreased *d*-spacing of rGO strengthened the fact of the removal of oxygen-bearing functional groups. In addition to this, the broad peaks suggested the amorphous nature along the stacking direction of rGO sheet and the presence of layers of rGO which were different from of crystalline graphite and GO. The X-ray diffraction pattern of PANI showed the diffraction peaks at 2*θ* = 14.7°, 20.9° and 25.0°. These peaks corresponded to the (011), (020) and (200) Bragg's reflections respectively which advocated the crystalline planes of emeraldine salt form of PANI.^35^ The XRD spectra of rGO–PANI nanocomposite corroborated the presence of only two characteristics peaks of rGO at 24.6° and 43.3°. The peaks of PANI were found to be completely disappeared due to its low content and noncrystalline nature. The XRD spectra of AgNPs–rGO–PANI revealed the presence of a characteristic peak of rGO and AgNPs confirming the AgNPs–rGO–PANI nanocomposite formation.

**Fig. 4:**
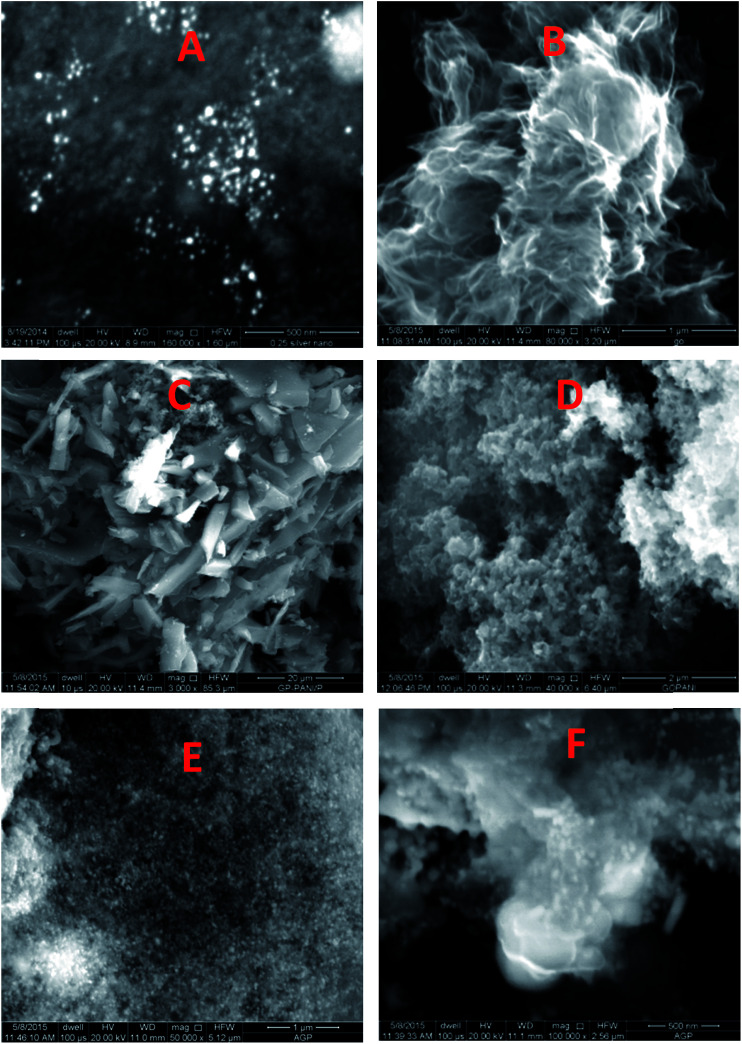
FE-SEM images of (A) AgNPs, (B), rGO, (C) PANI, (D) rGO–PANI, (E and F) AgNPs–rGO–PANI.

**Fig. 5:**
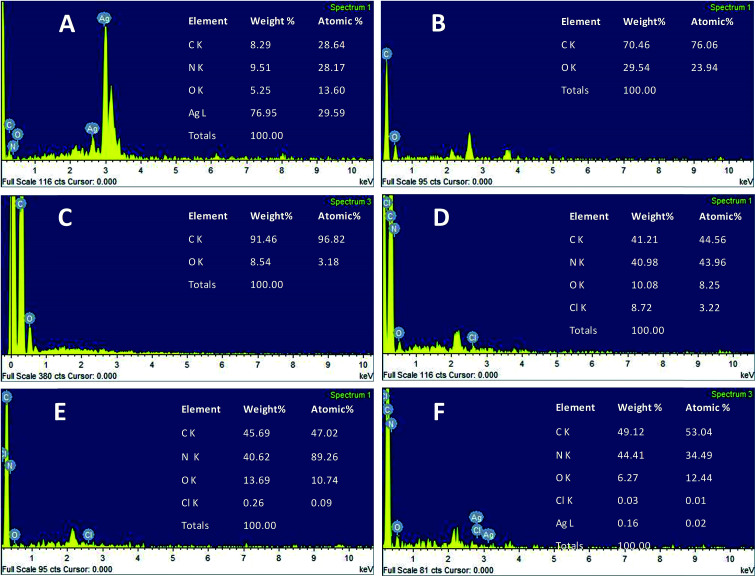
EDX spectrum of (A) AgNPs, (B) GO, (C) rGO, (D) PANI, (E) rGO–PANI, and (F) AgNPs–rGO–PANI.

The morphology of the AgNPs, rGO, PANI, rGO–PANI and AgNPs–rGO–PANI was investigated through FE-SEM analysis. Fig. 4A clearly showed that the spherical AgNPs were distributed throughout the mass of samples. The purity of AgNPs was analyzed using FE-SEM with Energy Dispersive X-ray detector (EDX). The EDX analysis produced a strong spectral signal of silver (Ag) at 3 keV which advocated the presence of silver nanocrystallites^27^ (Fig. 5A). The EDX analysis also corroborated the presence of distinct spectral signals for carbon and oxygen which corresponded to the biomolecules of AEC adhered at the surface of AgNPs. Fig. 4B showed FE-SEM image of rGO which confirmed the sheet-like structure of rGO. The corresponding EDX spectra of rGO shown in Fig. 5C revealed the presence of carbon and oxygen signals with 91.46 wt% and 8.54 wt% respectively whereas the EDX spectra of GO revealed 70.46 wt% carbon and 29.54 wt% oxygen (Fig. 5B). The decreased amount of oxygen (8.54 wt%) in rGO than GO (29.54 wt%) also advocated the reduction of GO to rGO.^21^Fig. 4C showed the flake-like structure of PANI which was advocated by the presence of a strong spectral signal for carbon, oxygen chlorine and nitrogen in the corresponding EDX spectra (Fig. 5D). FE-SEM image of rGO–PANI nanocomposite showed the distinct changes in morphology rather clear sheet of rGO deposited with PANI (Fig. 4D). It is obvious from the Fig. 5E that the characteristics spectral signals for rGO and PANI were present in the rGO–PANI nanocomposite. Fig. 4E and F showed the FE-SEM images of AgNPs–rGO–PANI which revealed the distribution of AgNPs at the surface of rGO–PANI showing the successful preparation of AgNPs–rGO–PANI nanocomposite which was advocated by the presence of the spectral signal of carbon, oxygen, chlorine, nitrogen and silver from the corresponding EDX (Fig. 5F).

**Fig. 6:**
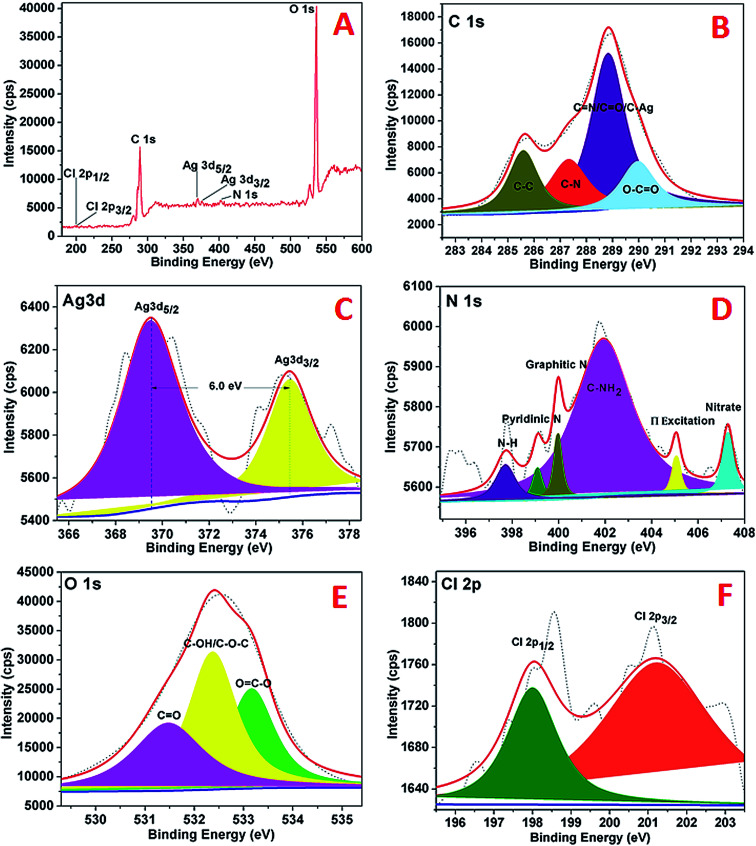
XPS spectra of AgNPs showing (A) wide scan, (B) C 1s spectrum, (C) Ag 3d spectrum, (D) N 1s spectrum, (E), O 1s, (F) Cl 2p.

The XPS analysis of the AgNPs–rGO–PANI was performed to examine the elemental composition and their different possible bonding interaction. The wide scan spectrum of AgNPs/rGO/PANI is shown in Fig. 6A. The wide scan spectrum represented the peaks corresponding to Cl 2p, C 1s, Ag 3d, O 1s and N 1s which depicted Cl, C, Ag, O and N as constituent elements present in the AgNPs–rGO–PANI nanocomposite. Curve fitting of the core level C 1s spectrum showed four Gaussian peaks at 285.59, 287.30, 288.8 and 289.98 eV which assigned to C–C, C–N, C=N/C=O/C–Ag, and O–C=O respectively (Fig. 6B). Fig. 6C depicted the Ag 3d core level spectrum of AgNPs. The Ag 3d spectrum of Ag corroborated two peaks at 369.50 and 375.40 eV which corresponded to the binding energies of Ag 3d_5/2_ and Ag 3d_3/2_ respectively. The splitting of the 3d doublet of Ag (5.90 eV), indicated the formation of metallic AgNPs (Ag^0^).^23^ The O 1s core level spectrum fitted into an individual component is represented in Fig. 6D which showed three peaks of the O 1s signal at 531.4, 532.3, and 533.1 eV. The peak at 531.4 eV indicated the presence of, C=O whereas the peaks at 532.3 eV and 533.1 eV represented to the C–OH/C–O–C and O=C–O respectively. The high-resolution curve fitted XPS spectrum of N 1s is shown in Fig. 9E. The spectra represented five peaks of N 1s centred at 397.0, 399.0, 401.0, 405.0, and 407.0 eV. The peak at 397.0 eV was due to the presence of –NH of PANI grafted at the rGO sheets. The peak centered at 399.9 eV advocated the pyridinic nitrogen attached with AgNPs whereas the peak at 401.0 eV suggested the graphitic nitrogen of rGO. The peak at 405.0 and 407.0 eV represented to the π excitation, and nitrate residue of AgNO_3_ salt used for AgNPs synthesis respectively. Fig. 6F represented the XPS spectrum of Cl 2p with two peaks at 198.02 eV and 201.2 eV which were due to the metal and organic chloride respectively.

### 3.2 Electrochemical characterization of AgNPs–rGO–PANI–GCE

#### 3.2.1 Cyclic voltammograms (CV)

**Fig. 7:**
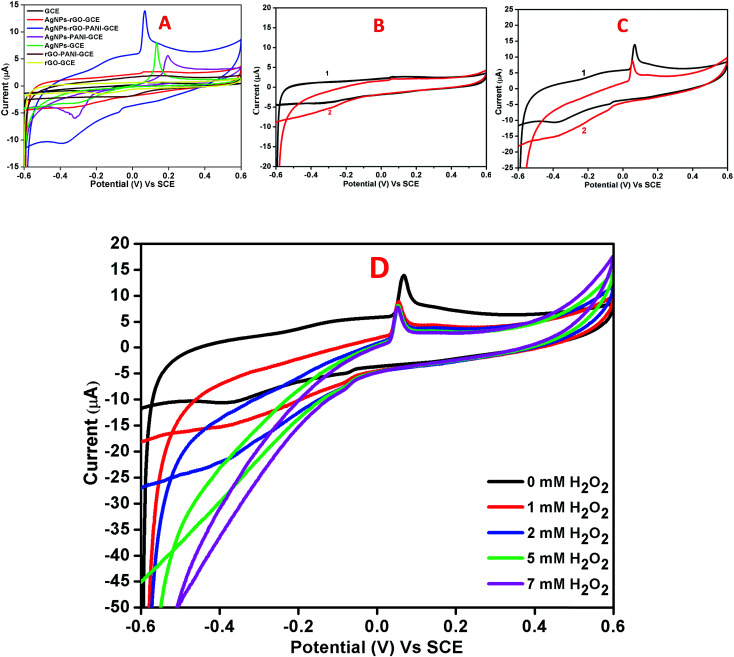
(A) Combined cyclic voltammetry of bare GCE, AgNPs–rGO–GCE, AgNPs–rGO–PANI–GCE, AgNPs–PANI–GCE, AgNPs–GCE, rGO–PANI–GCE, and rGO–GCE in 0.1 M phosphate buffer pH 7.0 containing 0.5 M KCl at 50 mV s^−1^ scan rate. Cyclic voltammograms of AgNPs–rGO–GCE (B) and AgNPs–rGO–PANI–GCE (C) in the absence (1) and presence (2) of 1 mM H_2_O_2_ in 0.1 M phosphate buffer pH 7.0 containing 0.5 M KCl at 50 mV s^−1^ scan rate. (D) CVs of AgNPs–rGO–PANI–GCE in 0.1 M phosphate buffer, pH 7.0 containing different concentrations of H_2_O_2_ (from 0 to 7 mM).

Fig. 7A showed the cyclic voltammograms of the bare GCE, AgNPs–rGO–GCE, and AgNPs–rGO–PANI–GCE carried out at a 50 mV s^−1^ scan rate and −0.6 to 0.6 V potential range in 0.1 M PBS (pH 7.0). The bare GCE did not show any significant redox whereas the AgNPs–rGO–GCE and AgNPs–rGO–PANI–GCE showed a distinct pair of redox peaks at the potential of 0.06 V and −0.4 V which corroborated the oxidation and reduction of AgNPs.^36–38^ As shown in Fig. 7B and C it was found that the reduction current increased for both the electrodes with the addition of 1 mM H_2_O_2_. However, the composite AgNPs–rGO–PANI–GCE showed a drastic increase in reduction current which indicated better electrocatalytic activity towards H_2_O_2_ than AgNPs–rGO–GCE (Fig. 7C). It was also observed that with the increase in the H_2_O_2_ concentrations from 1 mM to 7 mM, the peak current of AgNPs–rGO–PANI–GCE towards the reduction of H_2_O_2_ increased (Fig. 7D). The presence of PANI at the AgNPs–rGO–PANI–GCE favored the transfer of electron to H_2_O_2_ during electrocatalytic reduction. The major role played by the PANI is to facilitate larger surface area for the specific interaction with the targeted analyte (H_2_O_2_) so that it may enhance the electron-transfer kinetics during its electrocatalytic reduction. Such findings provided the valuable information on the use of AgNPs–rGO–PANI–GCE as an electrode modifier; the presence of PANI along with AgNPs–rGO–GCE character increased the analytical performance. Based on previously reported mechanism,^25,39,40^ the electrocatalytic reduction of H_2_O_2_ on electrocatalyst occurred according to the following mechanism:
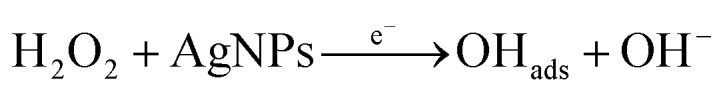

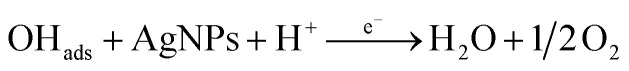


**Scheme 2:**
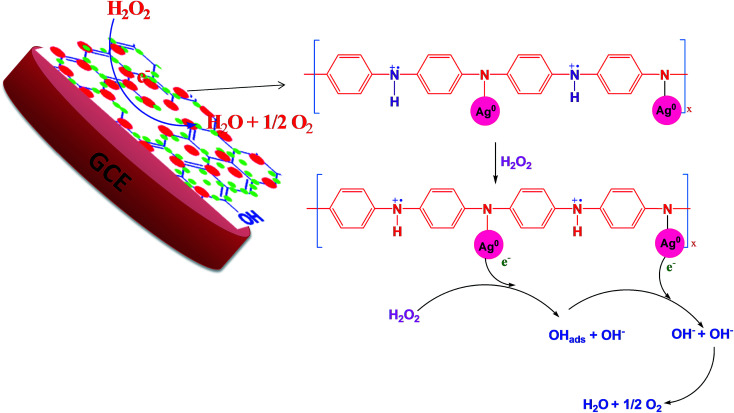
Schematic representation of the mechanism of H_2_O_2_ reduction.

According to the first equation, the adsorbed H_2_O_2_ on the AgNPs gained an electron from it and produced adsorbed OH (OH)_ads_ and OH^−^. After that, the (OH)_ads_ received another electron from AgNPs and produced H_2_O. Herein; the reduction rate depends mainly on two factors: (1) the adsorption of H_2_O_2_ at the surface of electrocatalyst and (2) transfer of an electron from electrocatalyst to (OH)_ads_. Therefore, the property of enhancement of adsorption and electron transfer of electrocatalyst is necessary for the electrocatalytic reduction of H_2_O_2_ (Scheme 2).

#### 3.2.2 Effect of scan rates

**Fig. 8:**
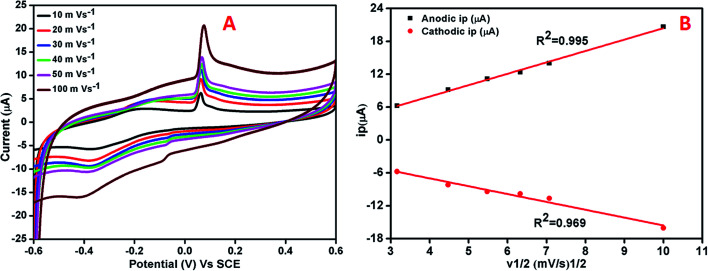
(A) Cyclic voltammetry curve of AgNPs–rGO–PANI–GCE at different scan rates in PBS [0.1 mM, pH 7.0, 0.5 M KCl], (B) linear variation of anodic and cathodic peak current *vs.* square root of scan rates.

The effect of varying scan rate on the potential and peak current was studied at AgNPs–rGO–PANI–GCE (Fig. 8A). The study did not show any change in oxidation and reduction peaks. The shifting of the oxidation and reduction peaks was observed towards the higher and lower potential respectively. This shifting was due to the transport of faster ion and electron between the interface of the AgNPs–rGO–PANI–GCE and electrolyte at the higher scan rate. The anodic and cathodic peak currents and the square root of scan rates from 10 to 100 mV s^−1^ showed a linear relationship with correlation coefficients (*R*^2^) of 0.99 and 0.96 respectively (Fig. 8B). This concluded that the reaction at the AgNPs–rGO–PANI–GCE was the surface controlled process.^41,42^

#### 3.2.3 Electrochemical impedance spectroscopy of AgNPs–rGO–GCE and AgNPs–rGO–PANI–GCE

**Fig. 9:**
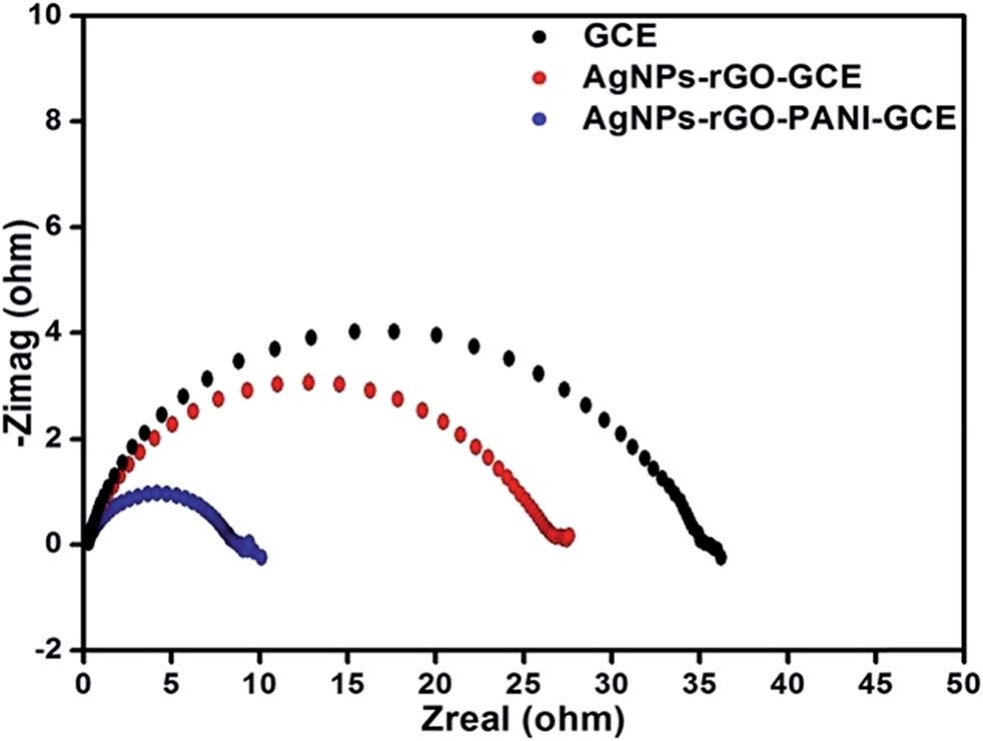
(A) Electrochemical impendence spectroscopy in PBS [0.1 mM, pH 7.0, 0.5 M KCl] of AgNPs–rGO, AgNPs–rGO–PANI modified electrode and bare GC electrode.

The EIS technique has been used for measuring the resistance of the modified electrode as a function of frequency because of variation in the interfacial properties. Fig. 9 showed the Nyquist plots of the AgNPs–rGO–GCE, AgNPs–rGO–PANI–GCE and bare GCE obtained in phosphate buffer [0.1 mM, pH 7.0, 0.5 M KCl]. The diameter of semicircle gives the value of charge transfer resistance (*R*_ct_), which intern reveals towards the electron-transfer kinetics occurring at the electrode interface. It is observed that the value of *R*_ct_ in case of AgNPs–rGO–PANI–GCE was lower (9 Ω) as compared to AgNPs–rGO and blank GCE (27.5 and 37 Ω), revealing that AgNPs–rGO–PANI–GCE given the more electroactive surface area and facilitated the electron transport between the medium and electrode.^43^

#### 3.2.4 Amperometric determination of H_2_O_2_

**Fig. 10:**
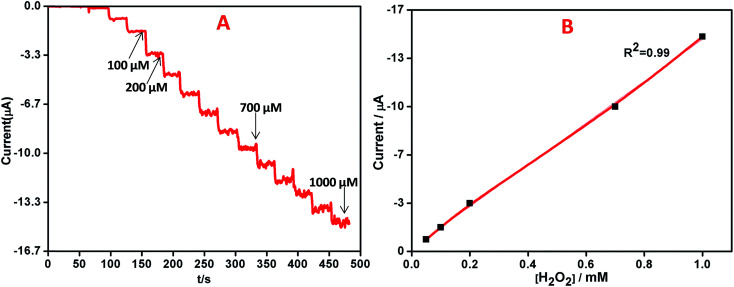
(A) Amperometric response of AgNPs–rGO–PANI–GCE on increasing the concentrations of H_2_O_2_ by 0.01, 0.02, 0.05, 0.1, 0.2, 0.5, 1, 2, 5, 10, 20, 50, 100, 200, 500, 1000 μM in 0.1 M PBS, pH 7.0 at −0.4 V *vs.* SCE, (B) the corresponding calibration curves for H_2_O_2_ analysis.

The quantitative determination of H_2_O_2_ was performed by recording the amperometric response on AgNPs–rGO–PANI–GCE by the addition of varying concentrations (0.01 μM to 1000 μM) of H_2_O_2_ in 0.1 M phosphate buffer (pH 7.0) at a working potential of −0.4 V *vs.* SCE. On the basis of the voltammetric results explained above, the amperometric measurement was performed to analyse the performance of AgNPs–rGO–PANI–GCE towards the electrochemical detection of H_2_O_2_, Fig. 10A showed a typical current–time plot for H_2_O_2_ detection on AgNPs–rGO–PANI–GCE by successive addition of H_2_O_2_ concentration. It was observed that the when the H_2_O_2_ was added into the stirring phosphate buffer, the response current was found to be increased rapidly and reached a steady state within 5 s which indicated a fast amperometric response towards the reduction of H_2_O_2_. The plot of current *vs.* concentration of H_2_O_2_ showed a linear relationship in the range of 0.01 to 1000 μM with a high correlation coefficient (*R*^2^ = 0.99) while the detection limitation was estimated to be 50 nM based on a signal-to-noise ratio (Fig. 10B). The sensitivity was calculated as 14.7 μA mM^−1^ cm^−2^, indicating a great potential as a non-enzymatic H_2_O_2_ sensor. The performance of AgNPs–rGO–PANI–GCE based H_2_O_2_ sensors was compared with the previously reported AgNPs based sensors as shown in Table 1. It was observed that the AgNPs–rGO–PANI–GCE had a wider linear range and lower detection limit. The lower detection limit obtained in present work was lower than, the lower detection limit obtained in most of the previous studies which showed good performance as a non-enzymatic amperometric sensor for H_2_O_2_.

**Table 1:** The table showing the comparison of previously reported analytical performance of non-enzymatic H_2_O_2_ sensors

Working electrode	Linear range (mM)	Detection limit (μM)	Reference
AgNPs/GCE	—	2	44
Ag nanowire–GCE	0.05–10.35	10	36
Ag–graphene–GCE	0.1–40	28	45
Ag NPs/3DG	0.03–16.21	14.9	25
GO–Ag/GCE	0.1–11	28.3	26
AgNPs–CNT–rGO/GCE	0.01–10	1	38
AgNPs/PANINFs	0.1–60	0.25	46
AgNPs/rGO/PANI	0.1–80	7.1	47
AgNPs–MWCNT–rGO/GCE	0.1–100	0.9	48
PpyNFs–AgNPs–rGO/GCE	0.1–5	1.099	49
	10–90	0.085	
AgNPs–rGO–PANI–GCE	0.01–1	0.05	Present work

#### 3.2.5 Interference study

**Fig. 11:**
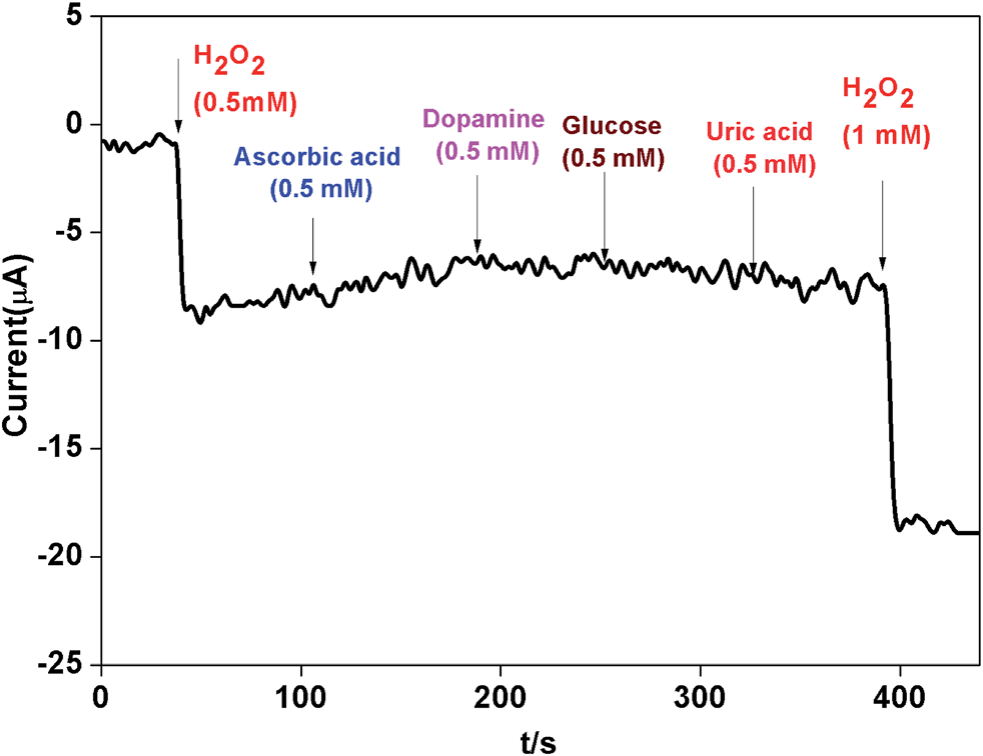
The amperometric response of AgNPs–rGO–PANI–GC electrode towards H_2_O_2_ and various interferential compounds (ascorbic acid, dopamine and glucose) in 0.1 M phosphate buffer (pH 7.0).

The influence from common interference species such as ascorbic acid (AA), dopamine (DA) and glucose was also investigated. Fig. 11 revealed that when AA, DA and glucose (0.5 mM) were added in 0.1 M phosphate buffer (pH 7.0) at a working potential of −0.4 V *vs.* SCE, the AgNPs–rGO–PANI–GCE did not show any amperometric response. Whereas when H_2_O_2_ (1 mM) was added it showed a strong amperometric response which confirmed the selectivity of the AgNPs–rGO–PANI–GCE towards H_2_O_2_.

## 4. Conclusions

In the present study, we have constructed the AgNPs–rGO–PANI nanocomposite as a simple electrochemical sensor towards the sensitive and selective detection of H_2_O_2_. Several instrumental techniques like UV-vis, FTIR, XRD, SEM, EDX, and XPS were used for the characterization purpose which advocated successful synthesis of AgNPs, GO, rGO, PANI, rGO–PANI and AgNPs–rGO–PANI nanocomposite. Thus obtained AgNPs–rGO–PANI nanocomposite was loaded (0.5 mg cm^−2^) on glassy carbon electrode (GCE) with 0.2 cm^2^ active surface area for the investigation of electrochemical properties. The AgNPs–rGO–PANI–GCE showed an excellent electrocatalytic performance on the reduction of H_2_O_2_. The amperometric current (μA) *vs.* time (s) curve displayed a linear range of 0.01–1000 μM (*R*^2^ = 0.99) with the low detection limit of 50 nM toward the electrochemical sensing of H_2_O_2_ which was due to the direct electronic interaction of AgNPs with N atom of the PANI backbone on the rGO sheet. This interaction enhanced the electron transfer kinetics during the electrochemical reduction of H_2_O_2_. High sensitivity, selectivity, wide linear range and low detection limit made the AgNPs–rGO–PANI–GCE as an excellent amperometric sensor for the detection of H_2_O_2_.

## Conflicts of interest

There are no conflicts to declare.

## Supplementary Material

RA-008-C7RA11466D-s001
