# Supplementary material for: Enhanced electron transfer mediated detection of hydrogen peroxide using a silver nanoparticle–reduced graphene oxide–polyaniline fabricated electrochemical sensor
Source: RSC Adv. 2018 Jan 4;8(2):619–31. doi: 10.1039/c7ra11466d (PMC9076931; doi:10.1039/c7ra11466d)
Supplement: RA-008-C7RA11466D-s001 [file RA-008-C7RA11466D-s001.pdf]

Electronic Supplementary Material (ESI) for RSC Advances.
This journal is © The Royal Society of Chemistry 2017

Figure S1

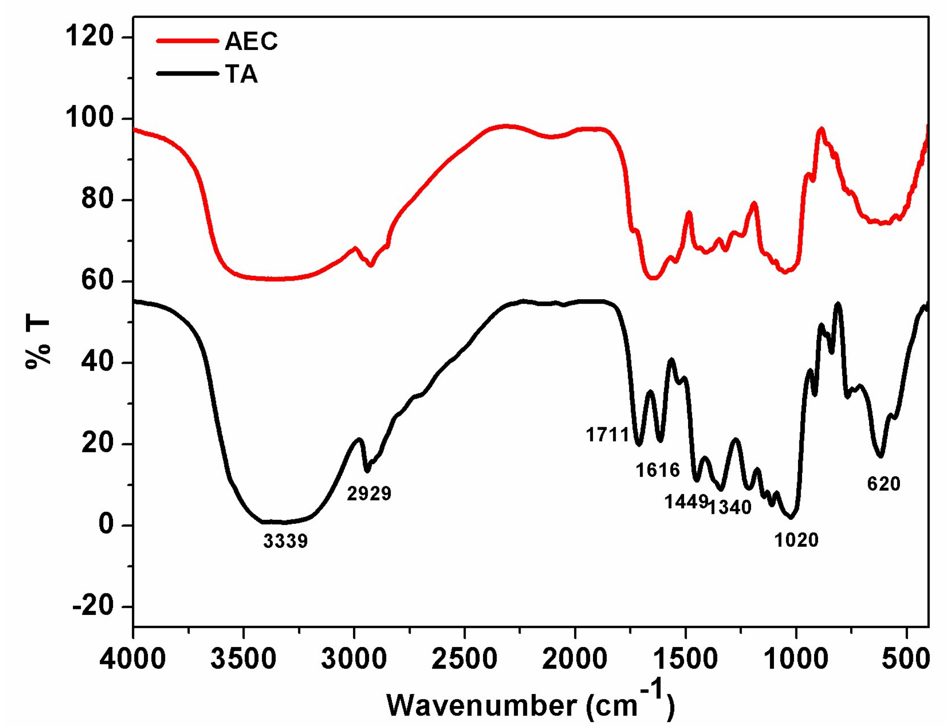

Figure S1 FTIR spectra of the tannic acid (TA) and aqueous extract of *Croton bonplandianum* (AEC)
